# Cardiac condition in young chagasic women

**Published:** 2013-06-25

**Authors:** O Lassen, G Dotto, S Ojeda, A Garutti, P Bertolotto, S Tabares, R Gallerano, A Sembaj

**Affiliations:** * Semiology Department UHMI 3, Chagas and Hypertension Office, Cordoba Hospital, Argentine; ** Central Laboratory of Cordoba Hospital, Córdoba, Argentine; *** School of Mathematics, Astronomy and Physics, UNC, Argentine; **** Biochemistry and Molecular Biology Department, School of Medicine, UNC, Cordoba, Argentine

**Keywords:** chagasic disease, EchoCG alterations, premenopausal women

## Abstract

Rationale: Chagas disease is a complex disorder caused by Trypanosoma cruzi. Most patients remain asymptomatic for several years and 30% of them progress quietly to developing cardiomyopathy. The factors that lead to chronic myocardial lesions are not fully understood.

Objective: To investigate the association between clinical symptoms and single nucleotide polymorphisms in chagasic and non-chagasic women younger and older than 55 years of age.

Methods and Results: we analyzed Ala-9Val and Ile58Thr polymorphisms of the SOD-Mn gene, 138ex1ins/del A of the endothelin-1 gene (ET-1) and H323H (T/C) of the endothelin receptor A gene (ETA), by PCR-RFLP using genomic DNA from leukocyte of 85 women. We also evaluated serum lipid profile, renal and liver function, chest X-rays, electrocardiograms (ECGs) and echocardiography (EchoCG). Biochemical profiling did not show differences between chagasic and non-chagasic patients. The polymorphisms analyses showed a significant association in the distribution of frequencies of the Mn-SOD Ile58Thr gene between both groups. Young chagasic patients had a significantly higher prevalence of abnormalities in X-rays, in ECGs and they showed grade II and III of NYHA functional classes. The chance of having an abnormal EchoCG was 5.87 higher in young chagasic patients (OR=5.87, 95% CI 1.47-23.4).

Discussion: We concluded that the parasite affects young females by accelerating the deterioration of cardiac function, independent of other cardiovascular risk factors and the cardioprotective action of estrogens present in the premenopausal stage.

## Introduction

Trypanosoma cruzi (T. cruzi) is the intracellular parasite responsible for Chagas disease, a severe health care problem in Latin America [**1**].The course of the infection is characterized by an acute phase followed by mild chronic infection and positive serology with low evidence of parasites and signs of disease. Approximately a third of the subjects develop chronic chagasic cardiomyopathy (CCC) several years after the primary infection. In Argentina, CCC causes an estimated 45,000 deaths every year [**2**]. One characteristic of Chagas disease is the difficulty in predicting who will develop symptoms. The variability of symptoms and disease evolution can be attributed to the existence of different strains of T. cruzi and to the specific genotype of each host [**3**]. The cardiomyopathies induce an overexpression of biologically active molecules, such as angiotensin and endothelin [**4**]. It has been described that increased serum level of endothelin-1 is associated in chagasic patients with the presence of cardiac involvement [**5**]. Some studies have shown that the polymorphism 138ex1ins/del A of the endothelin-1 gene is associated with cardiovascular compromise, enlargement of the left ventricle, hardening of the vascular endothelium, and hypertension in Japanese population [6,7]. The C/T polymorphism of exon 6 of the endothelin receptor A gene has been associated with survival prediction in patients with dilated cardiomyopathy [**8**]. Although it is clear that the clinical manifestations of cardiac diseases are due to the action of multiple genes, little is known about the association of polymorphisms with CCC. Cardiac cells, which have a high number of mitochondria, are susceptible to damage by oxygen, and consequently, Mn-dependent superoxide dismutase (Mn-SOD) plays an important role in protecting the heart against compounds that are partially oxidized [**9**]. Women report lower incidence of cardiovascular disease. It has been suggested that the cardioprotective effects of estrogens are accomplished through the increased synthesis of vasoactive mediators [**10**]. Different authors have studied the association between Chagas diseases and sex with controversial results. CCC is a common disorder in our population, and clinical signs of cardiovascular injury are heterogeneous. Women of reproductive and productive age constitute the group which is least affected by cardiovascular diseases. Little is known about the way chagasic infection affects premenopausal women. Therefore, we studied the association between cardiac conventional risk factors and certain polymorphisms with CCC in young chagasic women.

## Materials and Methods

 Case I

Population study: Eighty-five women between 30 and 78 years old, who attended to the Office of Chagas and Hypertension (Internal Medicine Department) of the Hospital Cordoba (Cordoba, Argentina), were recruited consecutively. All of them filled a questionnaire about the family history of cardiovascular diseases and underwent physical examination. The Ethics Committee of the Institution approved the study. All the subjects were treated according to the Helsinki declaration and signed a written informed consent before admission. Pregnant or hypertensive women were excluded. The subjects had a 12-lead electrocardiogram (ECG) at rest, transthoracic color Doppler echocardiography (EchoCG), and chest radiography (X-ray). Blood samples were obtained to perform routine biochemical tests and serological determination of Chagas disease. An aliquot was used to isolate DNA and perform subsequent analysis of single nucleotide polymorphisms (SNPs). The women considered as chagasic patients was diagnosed with 2 or more positive quantitative serological tests as indicated by the OPS [**11**]. The population was divided according to the cardiovascular assessment; they were classified as asymptomatic (without clinical, radiological, ECG and EchoCG indicators of cardiac lesions) or symptomatic, when at least one of these evaluations was abnormal. The subjects of each group were divided into premenopausal (<55 years old and plasma levels of estrogen 40-350 pg/mL) and postmenopausal (>55 years old and plasma levels of estrogen less than 30 pg/mL) groups. Women with estrogens levels out of the specified range were excluded. Genetic analysis Genomic DNA was extracted from peripheral white blood cells using conventional techniques [**12**]. The following SNPs related to endothelin axis were studied: insertion/deletion of adenine 138 (ins/del A) in the endothelin-1 gene (ET-1) [**6**], and cytosine (C) to thymine (T) 323 substitution in the endothelin-1 receptor A (ETA) gene [**8**]. In the Mn-SOD gene, we analyzed a T to C substitution that produces a valine 9 to alanine substitution (Ala9Val) [**9**]. We also examined Ile58Thr in exon 3 [**13**]. The polymorphisms were detected by PCR amplification and subsequent digestion with specific restriction enzymes (PCR-RFLP). After the digestion, the products were analyzed on a 3% agarose or 8% polyacrylamide gel electrophoresis stained with ethidium bromide. Positive and negative controls were used in each amplification reaction, and each sample was analyzed in duplicate. Statistical analysis Allele and genotype frequencies of the studied polymorphisms were obtained by direct counting. Hardy-Weinberg Equilibrium was calculated. Differences in biochemical and clinical variables between groups were assessed by using χ2 analysis or alternatively the two-sided Irwin Fisher test. P-values of less than 0.05 were considered statistically significant. Estimates with 95% confidence intervals (CI) were calculated as the odds ratio. Statistical analysis was performed by using SPSS version 15 (Chicago, Illinois, USA) and INFOSTAT.

## Results

The cardiac alterations determined by chest X-rays, ECGs and EchoCG showed that Chagasic women were more prone to have symptoms of Grade III of NYHA functional classes compared to non-chagasic women (59.3 and 35.3%, respectively, p=0.047). The biochemical results of the asymptomatic chagasic and non-chagasic women showed significant differences in the serum concentration of triglycerides and HDL cholesterol, 65% of chagasic postmenopausal females had normal values. The frequencies of abnormal chest X-rays, ECGs and EchoCG in each group are shown in Table 1.

**Table 1:** Frequencies of premenopausal and postmenopausal chagasic and non-chagasic women with or without heart abnormalities

	Cardiac Evaluation		Chest X-ray		ECGs		EchoCGs
		Normal% (n)	Abnormal% (n)	Normal % (n)	Abnormal% (n)	Normal % (n)	Abnormal% (n)
premenopausal	Non-chagasic	54 (14)	46 (12)	59 (16)	41 (11)	81(21)	19 (5)
	chagasic	31 (4)	69 (9)	29 (4)	71 (10)	43 (6)	57 (8) *
postmenopausal	Non-chagasic	20 (1)	80 (9)	20 (1)	80 (4)	50 (2)	50 (2)
	chagasic	9 (3)	91 (31)	9 (3)	90 (31)	12 (49)	88 (30)
Normal: with Rx Chest, ECG and EchoCG compatible with normalities. Abnormal: studies with some pathological alterations. Numbers indicate the percentage, in parentheses number of individuals,* is p<0.015;							

In general, premenopausal chagasic females showed a higher incidence of an abnormal chest X-ray, ECG, and EchoCG compared to premenopausal non-chagasic females. Chagasic premenopausal women were 5.87-fold more likely to have an abnormal EchoCG compared to premenopausal non-chagasic women (CI 1.47-23.39). Interestingly, the degree of heart damage was similar between both postmenopausal groups.

 In order to determine if the genetic composition of the subjects plays a role in the predisposition to develop more severe symptoms, we analyzed the SNPs of the endothelin-1 gene, its receptor A and two polymorphisms of the superoxide dismutase-Mn genes. We only observe a significant association in the distribution of frequencies of the Mn-SOD Ile58Thr gene polymorphism between both groups (p<0,0495). The group of patients with Chagas disease showed an excess of heterozygous (p<0.0167) for polymorphisms of ET-1 gene (+138/ex1ins/del A). In addition, for the polymorphism Ala-9Val of Mn-SOD gene, the population of non-chagasic patients showed an excess of heterozygous (p<0.0167). For the remaining loci, no deviation of the Hardy-Weinberg equilibrium was observed.

## Discussion

We have observed that young chagasic patients have various degrees of cardiomyopathy severity that are not usually detected in non-chagasic patients of the same age.

 Premenopausal and postmenopausal chagasic and non-chagasic females shared similar levels of normal or abnormal biochemical values. Therefore, these biochemical variables cannot explain the substantial differences in cardiac damage between chagasic and non-chagasic patients. Berra et al. [**14**] studied a population of males and females comparing chagasic and non-chagasic patients and observed that certain risk factors, such as smoking, alcoholism, obesity, and hypertension, were not significantly associated with increased risk of cardiac disease in chagasic people [**14**]. Therefore, in young chagasic patients, the T. cruzi is responsible for cardiac damage rather than traditional cardiovascular disease risk factors. 

 In our population, we observed a significant association between chagasic and non-chagasic patients and the MnSOD Ile58Thr gene polymorphisms. It is known that the Ile56Thr amino acid exchange affects the stability at the tetramer interface of MnSOD and reduces its enzymatic activity. Several studies demonstrate that the oxidative stress is associated with Chagas cardiomyopathies, the plasmatic SOD activity decline from 192,4±45.7 ng/mg blood protein in healthy group to 82.8 ±33.11 ng/mg blood protein in plasma of chagasic patients [**15**]. However, we did not observe an association between cardiac symptoms and the gene polymorphisms analyzed in this work.

 According to our observations, estrogens levels do not protect against the pathologic process generated by T. cruzi infection, because young chagasic women develop myocardial damage long before menopause; moreover, in non-chagasic patients, myocardial damage usually takes place after menopause. The aggressiveness of T. cruzi is evidenced by the severe decline in systolic function observed on two-dimensional EchoCG (EF <40% and grade III dilation of the cardiac cavities).

 We did not observe statistical differences between the ECGs of chagasic and non-chagasic patients, even though the incidence of premenopausal chagasic patients with an abnormal ECG is much higher. After menopause, the incidence of patients with an abnormal ECG reaches the same values in both groups. In non-chagasic patients, this is likely because of the complications generated by hypertension, and in chagasic patients, this is likely because of the long time since the original lesion manifested.

 Our observations do not seem to be influenced by anthropometric or clinical factors because experiments of both groups were strictly controlled for confounding variables (smoking, sedentary lifestyle, overweight, among others). The biochemical variables that indicate a risk of developing cardiomyopathy are not substantially different between the two groups of premenopausal women. The difference in the incidence of patients with abnormal EchoCG seems to be due to mechanisms that are activated by T. cruzi infection. Because the young women are considered a group with low risk of developing cardiac injuries, a close clinical follow up and early identification of cardiac alterations in young chagasic women is necessary.

Acknowledgments

 This work was supported by a grant 05/H217 from Secretaria de Ciencia y Tecnología de la Universidad Nacional de Córdoba (SECyT-UNC). Córdoba, Argentina
